# Bowel habits and gender correlate with colon length measured by CT colonography

**DOI:** 10.1007/s11604-021-01204-7

**Published:** 2021-10-11

**Authors:** Kenichi Utano, Koichi Nagata, Tetsuro Honda, Takashi Kato, Alan Kawarai Lefor, Kazutomo Togashi

**Affiliations:** 1 Department of Coloproctology, Aizu Medical Center, Fukushima Medical University, 21-2 Maeda, Tanisawa, Kawahigashi, Aizuwakamatsu, Fukushima 969-3492 Japan; 2 Department of Gastroenterology, Fukushima Medical University, Fukushima, Japan; 3 Department of Gastroenterology, Nagasaki Harbor Medical Center, Nagasaki, Japan; 4 Department of Internal Medicine, Hokkaido Gastroenterology Hospital, Sapporo, Japan; 5 Department of Surgery, Jichi Medical University, Shimotsuke, Japan

**Keywords:** Computed tomographic colonography, Gender, Constipation, Defecation

## Abstract

**Purpose:**

CT colonography enables three-dimensional measurement of colon length. However, previous studies using CT colonography have not examined the association with gender, age, physique, a history of laparotomy and bowel habits, all possible contributory factors to colon length. The aim of this study is to investigate factors associated with colon length.

**Materials and methods:**

We conducted a post hoc analysis based on data obtained from a previous multi-center trial including 321 patients with positive fecal immunochemical tests who underwent CT colonography. Colon length was measured using a computer-generated center line and was divided at the iliac crest level into the distal and proximal colons. Bowel habits were classified into three groups: A—daily; B—once every 2 or 3 days; and C—less than once in 3 days. Statistical comparison was made using one-way ANOVA with Bonferroni’s correction.

**Results:**

A total of 295 patients were analyzed. The entire colon length (cm, mean ± standard deviation) of individual patients was 150.3 ± 18.5 cm and ranged from 109.7 to 195.9 cm. The female colon was significantly longer than the male colon (154.3 ± 18.1 cm vs. 147.1 ± 18.3 cm; *p* = 0.022). Colon length showed trends associated with age (*p* = 0.18) and a history of laparotomy (*p* = 0.14). According to bowel habits, the entire colon measured 147.4 ± 17.9 in group A, 154.7 ± 18.5 in group B and 158.6 ± 18.3 in group C, and significant differences were observed for “A vs. C” (*p* = 0.002) and “A vs. B” (*p* = 0.014). In subgroup analysis by colon segment, the proximal colon trended similarly to the entire colon while there were no trends for the distal colon.

**Conclusions:**

This study has clearly demonstrated that bowel habits and gender both correlate with the length of the entire colon measured by CT colonography, and in particular, the proximal colon.

**Secondary abstract:**

Using CT colonography, we measured the colon length in 295 patients. The entire colon length was 150.3 ± 18.5 cm on average. Females and constipated (less frequent defecation) patients have a significantly longer colon, and in particular, the proximal colon. Colon length showed trends associated with age and a history of laparotomy.

**Supplementary Information:**

The online version contains supplementary material available at 10.1007/s11604-021-01204-7.

## Introduction

Constipation is one of the most common chronic gastrointestinal conditions, which reportedly affects 14–17% of the population [1, 2], more frequently in females and the elderly [3], and adversely affects both physical and psychological quality of life [4]. In general, endoscopists have empirical knowledge that difficult colonoscopy is frequently encountered in elderly, thin or female patients, or who have constipated bowel habits, a history of laparotomy, or diverticular disease [5, 6], and that difficult colonoscopy is mostly attributed to a long and redundant colon [7].

Since the nineteenth century, there have been many studies of the anatomy of the large intestine at autopsy [8–10]. These cadaver studies attempted to elucidate the association of colon length with age, gender and physique, and showed inconsistent results probably due to varying degrees of postmortem rigidity after death. In 1995, Saunders et al. reported variations in colon length in 118 patients undergoing laparotomy, measuring according to a set protocol with the bowel pulled medially, mimicking the possible displacements that may occur during colonoscopy [11]. However, this measurement method possesses intrinsic limitations, e.g., inability to measure a colon with adhesions or a distal rectum, resulting in a shorter colon than using other methods. Barium enema examination allows two-dimensional estimation of the colon length mainly using an opisometer (mapping wheel). Despite the rough estimates, this measurement method found a correlation of colon length with colon transit time [12], difficult colonoscopy [13], incidence of sigmoid volvulus [14], age and gender [15]. Colonic transit time, that is, an objective test to diagnose slow transit constipation and other gastrointestinal symptoms correlated with colon redundancy rated in a non-quantitative manner [12]. In another study to investigate colon anatomy and motility in young women with severe idiopathic constipation [16], the colon was longer in constipated women (*n*=37) than in controls (*n*=20), but the difference was not significant probably due to insufficient statistical power of the study.

With the recent emergence of computed tomography (CT) colonography, three-dimensional measurement of colon length is feasible. This means that physicians can easily obtain information on the actual colon length of live patients. So far, several researchers have measured colon length using CT colonography to identify the cause of incomplete colonoscopy [17], colon length difference compared with colonoscopy [18, 19], positional alteration of the colon length [20] and the correlation of colon anatomy with age, gender and body mass index [21]. Ohgo et al. reported that the entire colon in patients with functional constipation was significantly longer than patients with diarrhea-type irritable bowel syndrome (IBS) or control patients in a small-scale case–control study [22]. However, previous studies using CT colonography have not examined the association with gender, age, physique, a history of laparotomy and bowel habits, all possible contributory factors to colon length. The aim of this study was to comprehensively investigate factors which correlate with colon length in a large observational cohort, particularly focused on bowel habits.

## Materials and methods

### Study design

This study adopted a post hoc design, using data obtained from a previous multi-center trial (UMIN Clinical Trials Registry number UMIN000006665) including 321 patients with positive fecal immunochemical tests (FIT) who underwent CT colonography [23], which was performed in seven hospitals across Japan. Ethical review approval was obtained from all seven hospitals and all patients gave their written informed consent, including for this post hoc analysis.

### Patients

Patients were eligible for inclusion into the study if they were 40 years of age or older and were seen at participating institutions to undergo colonoscopy for colorectal cancer examination because of a recent positive FIT result. Patients were excluded for any of the following reasons: serious comorbidity; a history of colon screening tests in the preceding 3 years; known colorectal neoplasms; a history of inflammatory bowel disease, Lynch syndrome, familial polyposis, or colorectal surgery; pregnancy, hyperthyroidism, or iodinated contrast medium allergy. Eligible consecutive patients were prospectively enrolled in the study between December 2011 and September 2012.

Prior to enrollment, information on bowel habits was obtained through an interview by nursing staff. Bowel habits were classified into three categories according to the frequency of bowel movements: daily group, every day; intermediate group, once every 2 or 3 days; and constipated group, less than once in 3 days. Patients’ information on age, gender, height, weight, body mass index (BMI) and history of laparotomy were also obtained. Patients status-post gastrointestinal or uterus resection were classified as major, and those without such resections (e.g., appendectomy) were classified as minor laparotomies.

### CT colonography

Preparation started 1 day before CT colonography, and, patients received 380 mL polyethylene glycol solution (Niflec; Ajinomoto Pharmaceuticals, Japan), 20 mL iodinated oral contrast agent for fecal tagging, and two doses of 20 mg mosapride (Gasmotin; Sumitomo Dainippon Pharma, Japan) the day before CT colonography after breakfast and dinner. On the examination day, patients were placed in the left decubitus position for thin flexible rectal-tube insertion with a balloon. The colon was insufflated using an automated carbon dioxide insufflator prototype (HP-2, Horii Pharmaceutical Ind, Japan) and insufflation pressure was set to 20 mmHg. The total volume of carbon dioxide insufflated was recorded. All CT colonography examinations at all institutions were performed using 16- or 64-row multi-detector CT scanner, with supine and prone positioning. The scanning protocol was as follows: 120 kVp, automatic tube current modulation system or a tube current of 50 mA, and a section thickness ≤ 1.0-mm. Spasmolytic agents and intravenous contrast media were not used during CT colonography [24].

### Image analysis

All CT colonography images were analyzed by an experienced radiologist who had over 10 years of abdominal diagnostic radiology experience. As part of image processing, the workstation automatically creates a centerline for 3D fly-through image review. We recorded the distance of the centerline from the anus to the cecal tip. All interpretations were performed using a commercially available workstation (AZE Virtual Place, Canon Medical System, Japan). Colon length was measured in two positions: the supine position and the prone position, and the average of the two measurements was defined as the colon length of each subject. The entire colon length was the distance from the anus to the cecal tip, and the colon was divided into two parts (proximal colon: cecum to descending colon, distal colon: sigmoid colon and rectum). The boundary between the descending colon and sigmoid colon was at the height of the iliac crest.

### Outcome measurements

The primary outcome is the mean entire colon length of the two measurements in the supine and prone positions, by age group (40–59, 60–79, 80 years or older), gender, body mass index (underweight: − 18.5, normal weight: 18.5–25, overweight: 25–), history of laparotomy (major, minor, none) and bowel habits (daily, intermediate, constipated). Secondary outcomes are proximal colon length and distal colon length, using the same factors mentioned above.

### Statistical analysis

The skewness and kurtosis test was used to test for a normal distribution of colon length. If normal distribution is confirmed, colon length is expressed as mean ± standard deviation (SD) and compared using one-way ANOVA with Bonferroni’s correction for multiple comparisons. Otherwise, colon length is expressed as median [interquartile range] and compared using nonparametric tests. A multivariate linear regression model was applied to analyze factors correlating with colon length. Categorical variables were presented as percentages and numbers and were compared by the *χ*^2^ test or Fisher exact test if appropriate. The threshold for significance was *p* < 0.05. All statistical analyses were performed with Intercooled Stata 16.0™ for Windows (Stata Corp. USA).

## Results

### Study patients

**Fig. 1:**
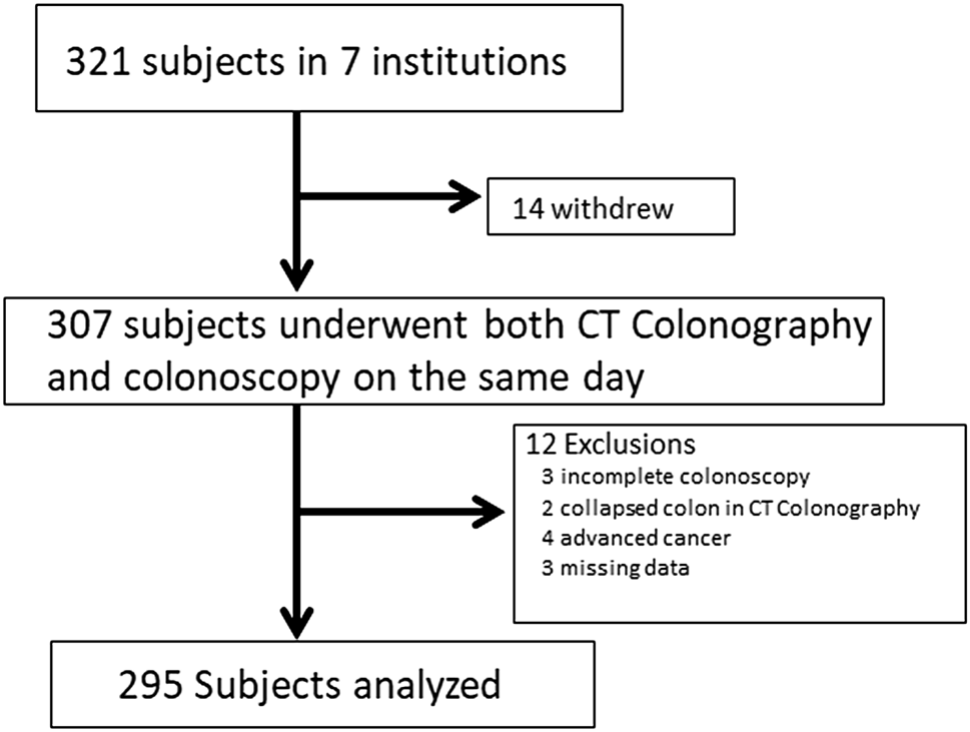
Study flow diagram. *CTC* computed tomography colonography

After excluding patients with poor bowel preparation, insufficient insufflation of the colorectum during CT colonography acquisition, or with a history of bowel resection and advanced cancer (stage T2: proper muscle layer or beyond), 295 patients (mean age 58.0 ± 11.0 years, range 40–83 years; female 141) were enrolled (shown in Fig. 1). The average total volume of carbon dioxide insufflated was 2612 mL (range: 1200–6900 mL). The characteristics of the patients are summarized in Table 1. The characteristics of patients by gender is shown in Supplementary Table 1. Height, weight and BMI were significantly greater in male patients, compared with females. Females had a significantly higher proportion undergoing major surgery and having constipated bowel habits. Bowel habits classified according to defecation frequency revealed 64% in the daily group, 23% in the intermediate group and 13% in the constipated group. Characteristics of patients by bowel habits are shown in supplementary Table 2. Age, BMI and a history of laparotomy have no significant differences. In contrast, the proportion of patients with “daily” bowel habits was significantly lower in females compared with males (“daily” vs. “intermediate”: *p* < 0.001; “daily” vs. “constipated”: *p* < 0.001). Patients with “daily” bowel habits had a trend toward higher weight (vs. “intermediate”: *p* = 0.077; vs. “constipated”: *p* = 0.01) and significantly greater height compared to other bowel habit patterns (vs. “intermediate”: *p* = 0.036; vs. “constipated”: *p* = 0.001).

**Table 1:** Study patients’ characteristics

Gender	
Male, *n* (%)	154 (52%)
Female, *n* (%)	141 (48%)
Age, years	
Mean ± SD	58.0 ± 11.0
40–59, *n* (%)	163 (55%)
60–79, *n* (%)	121 (41%)
80 or older, *n* (%)	11 (3%)
Weight, kg	
Mean ± SD	60.2 ± 11.1
Height, cm	
Mean ± SD	161.4 ± 8.7
Body mass index, kg/m^2^	
Mean ± SD	23 ± 3.3
History of laparotomy	
Major, *n* (%)	15 (5.1%)
Minor, *n* (%)	72 (24%)
None, *n* (%)	208 (71%)
Bowel habits	
Daily, *n* (%)	189 (64%)
Intermediate, *n* (%)	68 (23%)
Constipated, *n* (%)	38 (13%)

Bowel habits were divided into three groups by defecation frequency: daily group, every day; intermediate group, once every 2 or 3 days; and constipated group, less than once in 3 days. History of laparotomy: those who underwent gastro-intestinal or uterus resections were classified as major and others were classified as minor

*SD* standard deviation

### Colon length and associated factors (shown in Table 2)

**Table 2:** Colon length by variables correlating with colon length

	*N*	Entire colon length, cm (mean ± SD)	*p* value*	Proximal colon length, cm (mean ± SD)	*p* value*	Distal colon length, cm (mean ± SD)	*p* value*
All	295	150.3 ± 18.5		90.1 ± 12.9		60.4 ± 11.3	
Age (years)							
40–59	163	149.6 ± 17.2	0.18	89.2 ± 12.2	0.10	60.5 ± 10.8	0.74
60–79	121	150.9 ± 19.5		90.7 ± 13.4		60.1 ± 11.9	
80 or older	11	160.2 ± 25.2		97.3 ± 17.9		62.9 ± 13.4	
Gender							
Male	154	147.1 ± 18.3	0.022	87.5 ± 12.9	0.0003	59.5 ± 11.5	0.17
Female	141	154.3 ± 18.1		92.9 ± 12.5		61.4 ± 11.1	
Body mass index							
< 18.5	18	151.3 ± 18.7	0.73	90.2 ± 15.8	0.17	61.1 ± 13.1	0.64
≥ 18.5, < 25	201	150.0 ± 18.2		89.3 ± 11.8		60.7 ± 11.2	
≥ 25	74	151.9 ± 19.6		92.6 ± 14.9		59.3 ± 11.3	
History of laparotomy							
Major	15	156.7 ± 17.5	0.14	96.7 ± 14.3	0.051	59.9 ± 10.8	0.56
Minor	72	149.7 ± 17.5		87.9 ± 11.8		61.7 ± 9.5	
None	208	150.4 ± 18.9		90.4 ± 13.1		60.0 ± 11.9	
Bowel habits							
Daily	189	147.7 ± 17.9	0.003	88.2 ± 12.6	0.001	59.2 ± 11.1	0.058
Intermediate	68	154.7 ± 18.5		92.0 ± 12.8		62.7 ± 12.0	
Constipated	38	158.6 ± 18.3		96.3 ± 12.8		62.2 ± 10.5	

Colon was divided into proximal segment (cecum to descending colon) and distal segment (sigmoid colon and rectum) at the height of the iliac crest. History of laparotomy was classified in major (e.g., gastro-intestinal or uterus resection), minor (e.g., appendectomy) and none. Bowel habits were classified into a daily group (every day), an intermediate group (once every 2 or 3 days) and a constipated group (less than once in 3 days)

*SD* standard deviation

*Calculated using one-way ANOVA

**Fig. 2:**
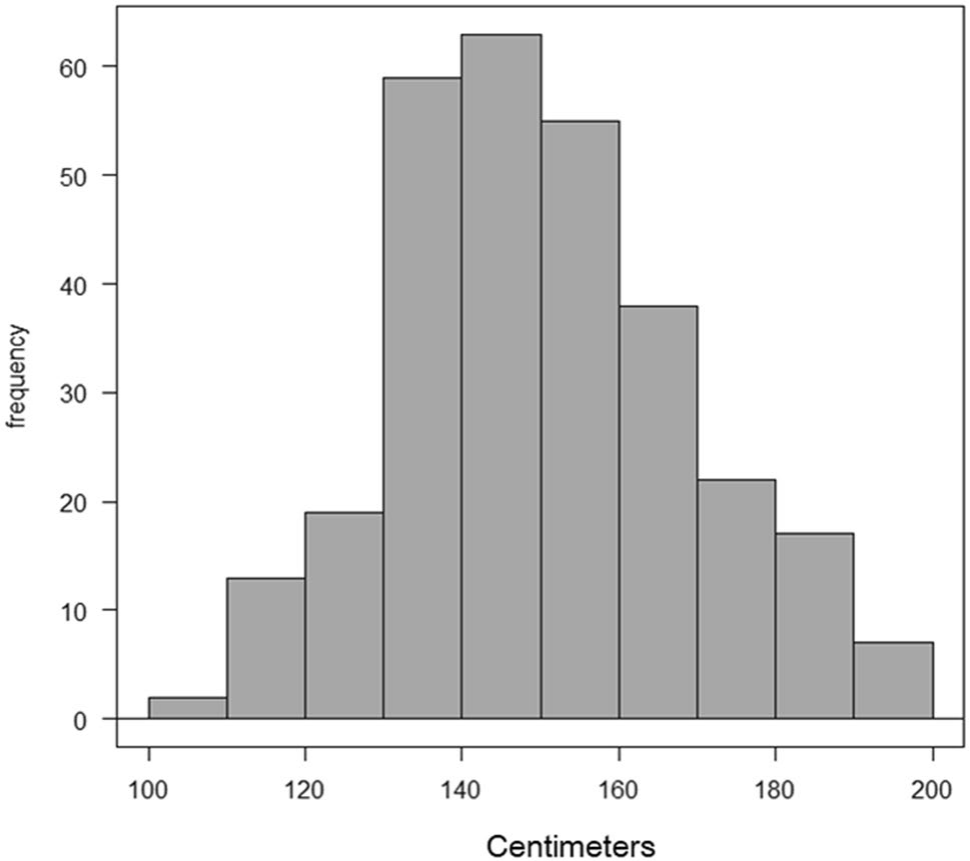
Histogram of mean entire colon length: the length of the entire colon had a normal distribution, which was confirmed by a skewness and kurtosis test (*p* = 0.104)

**Fig. 3:**
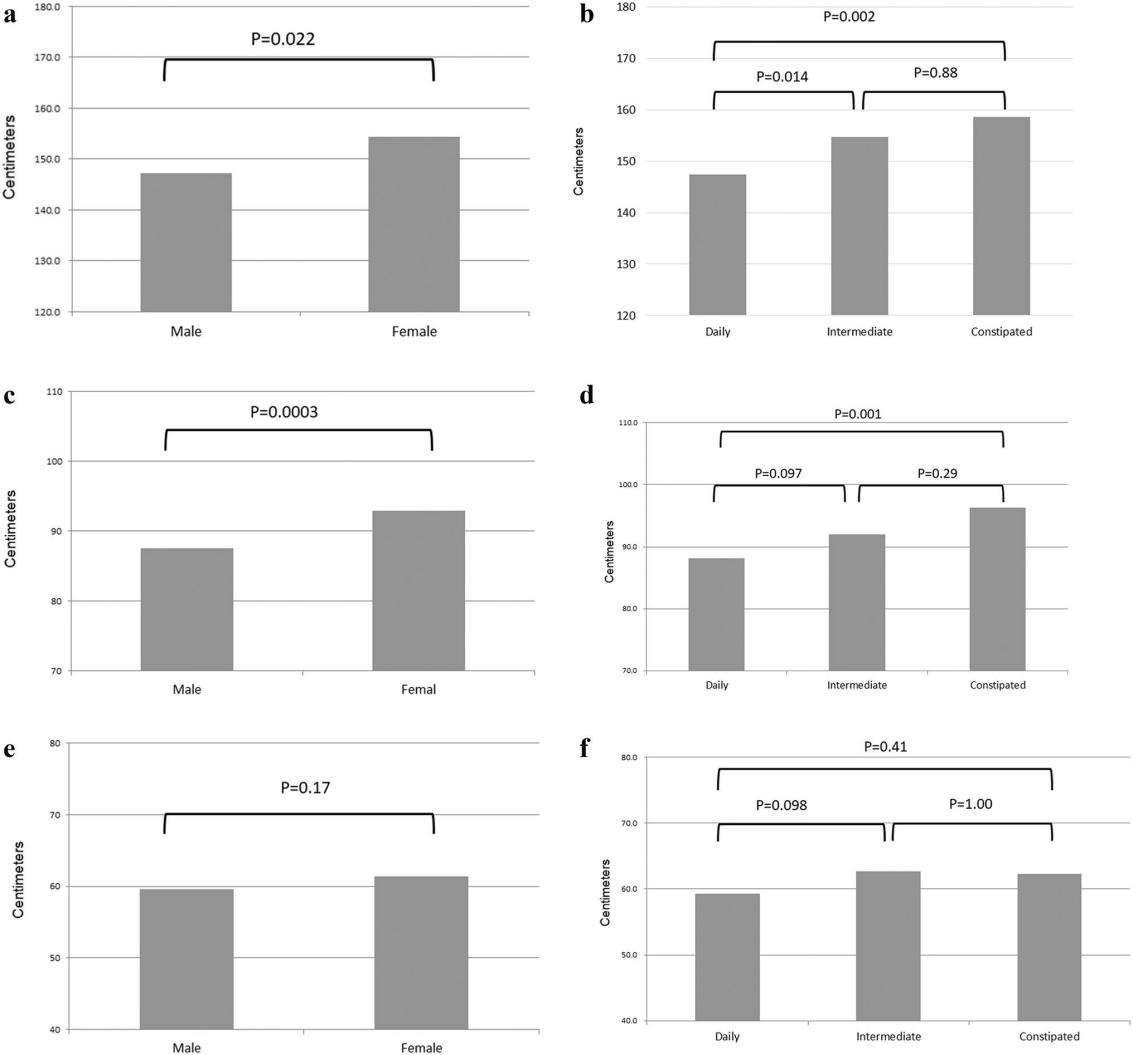
**a** Entire colon length by gender. **b** Entire colon length by bowel habits. **c** Proximal colon length by gender. **d** Proximal colon length by bowel habits. **e** Distal colon length by gender. **f** Distal colon length by bowel habits

**Fig. 4:**
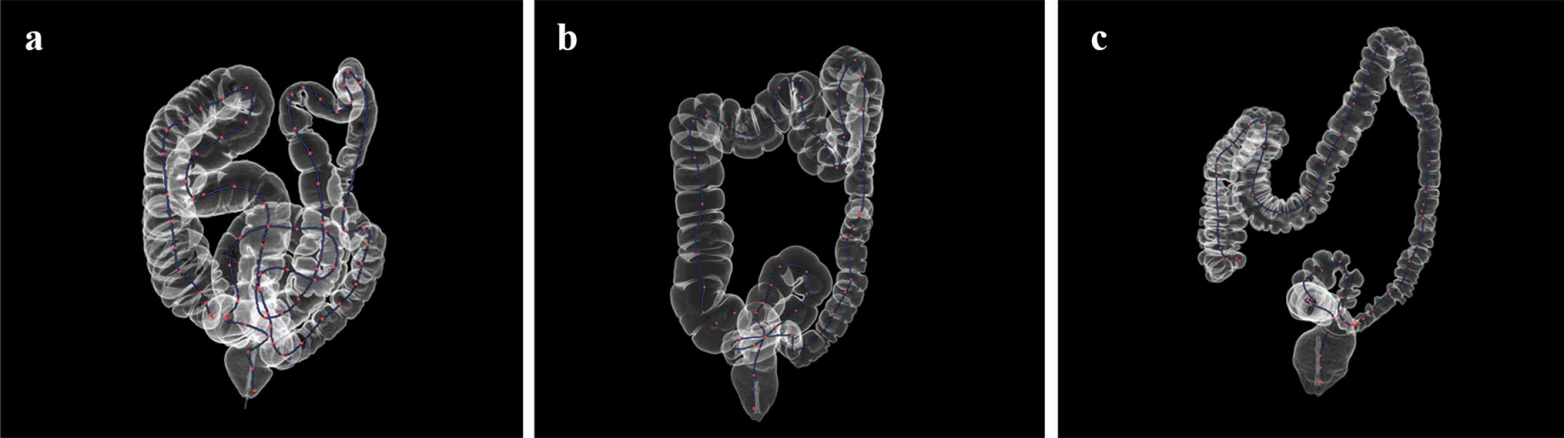
**a** Constipated group (defecation: less than once in 3 days). 65 years old, female, body height 151 cm, body weight 48 kg, BMI 21.0. The colon is long and redundant (entire colon 190.9 cm, proximal colon 114.6 cm, distal colon 76.3 cm, in the supine position). **b** Intermediate group (defecation: once every 2 or 3 days). 53 years old, female, body height 154 cm, body weight 55 kg, BMI 23.2. The colon looks slightly long (entire colon 152.2 cm, proximal colon 82.9 cm, distal colon 69.3 cm, in the supine position). **c** Daily group (defecation: every day). 61 years old, male, body height 177 cm, body weight 57 kg, BMI 18.1. The colon looks short (entire colon 115.4 cm, proximal colon 67.7 cm, distal colon 47.7 cm, in the supine position)

The length of the entire colon had a normal distribution (shown in Fig. 2), which was confirmed by a skewness and kurtosis test (*p* = 0.104). The entire colon length (mean ± SD) of individual patients was 150.3 ± 18.5 cm and ranged from 109.7 to 195.9 cm. The entire colon of the “80 years or older” age group had a trend toward being longer, but significant differences were not observed in any comparisons (vs. “40–59”: *p* = 0.203; vs. “60–79”: *p* = 0.335). In contrast, the female colon is significantly longer than the male colon (*p* = 0.022, shown in Fig. 3a). Any group categorized according to BMI had almost the same colon length. The “major” laparotomy group had a trend toward a longer colon, but there were no significant difference among any comparisons. Colon lengths by bowel habits were significantly different (*p* = 0.003, shown in Fig. 3b). The entire colon in the constipated group was significantly longer than in the daily group (*p* = 0.002), but not significantly different from the intermediate group (*p* = 0.877). The colon in the intermediate group was significantly longer than in the daily group (*p* = 0.014). Examples according to bowel habits are shown in Fig. 4a, b, c.

In subgroup analysis by segment of colon (shown in Table 2), the proximal colon trended similarly as the entire colon (shown in Fig. 3c, d), but there were no trends observed for the distal colon (shown in Fig. 3e, f). The female proximal colon is significantly longer than the male proximal colon (*p* = 0.0003). The proximal colon in the constipated group was significantly longer than in the daily group (*p* = 0.001). The proximal colon of patients undergoing major surgery had a trend toward being longer but only comparison between major and minor previous laparotomies approached a statistically significant difference (*p* = 0.051).

In subgroup analysis by gender (Table 3), female colon length by bowel habits was significantly different (*p* = 0.022). The female entire colon in the constipated group was significantly longer than in the daily group (*p* = 0.026), but there was no significant difference between the constipated group and intermediate group (*p* = 0.93) and between the intermediate group and daily group (*p* = 0.25). In contrast, significant differences were not observed in any comparisons in male colon (*p* = 0.26).

**Table 3:** Colon length by gender and bowel habits

	Male				Female			
	Daily	Intermediate	Constipated	*p* value*	Daily	Intermediate	Constipated	*p* value*
Entire colon Length (cm)	145.8 ± 18.3	151.9 ± 17.1	150.9 ± 17.3	0.26	150.2 ± 16.6	156.3 ± 18.9	160.6 ± 17.7	0.022*
Proximal colon length (cm)	86.7 ± 13.1	90.8 ± 11.1	90.0 ± 13.4	0.31	90.8 ± 11.3	92.7 ± 13.5	98.0 ± 11.9	0.021*
Distal colon length (cm)	59.1 ± 11.7	61.2 ± 11.8	60.9 ± 7.1	0.68	59.5 ± 10.0	63.5 ± 12.0	59.5 ± 10.0	0.130

Proximal colon: cecum to descending colon. Distal colon: sigmoid colon and rectum. Bowel habits were classified in a daily group (every day), an intermediate group (once every 2 or 3 days) and a constipated group (less than once in 3 days)

*Calculated using one-way ANOVA

### Linear regression analysis of factors associated with colon length (shown in Table 4)

**Table 4:** Multivariate linear regression analysis for variables correlating with colon length

	Entire colon			Proximal colon			Distal colon		
	Coefficients	95% CI	*p* value	Coefficients	95% CI	*p* value	Coefficients	95% CI	*p* value
Age	0.22	0.00 to 0.45	0.053	0.19	0.03 to 0.35	0.017	0.031	− 0.11 to 0.17	0.66
Gender (M 1, F 2)	10.8	3.9 to 17.6	0.002	8.8	4.0 to 13.5	< 0.001	2.0	− 2.3 to 6.3	0.37
Weight	− 0.0041	− 2.0 to 2.0	0.997	− 0.57	− 0.36 to 1.80	0.42	0.57	− 0.71 to 1.84	0.38
Height	0.38	− 1.2 to 1.9	0.63	0.72	− 0.90 to 0.61	0.19	− 0.34	− 1.33 to 0.65	0.50
Body Mass Index	0.40	− 4.9 to 5.7	0.88	2.0	− 1.7 to 5.6	0.29	− 1.6	− 4.9 to 1.8	0.35
History of surgery	0.057	− 3.6 to 3.8	0.98	− 0.78	− 3.35 to 1.80	0.55	0.83	− 1.51 to 3.18	0.49
Bowel habits	4.7	1.6 to 7.7	0.003	2.9	0.8 to 5.1	0.007	1.7	− 0.2 to 3.7	0.079

Colon was divided into proximal segment and distal segment at the height of the iliac crest. History of laparotomy was classified in major (2), minor (1) and none (0). Bowel habits were classified into a daily group (1), an intermediate group (2) and a constipated group (3)

*CI* confidence interval, *M* male, *F* female

In multivariate linear regression analyses of the length of the entire colon, significant differences were observed for gender (*p* = 0.002) and bowel habits (*p* = 0.003). Age approached statistical significance (*p* = 0.053). In analyses of the length of the proximal colon, similar trends were observed, and age was significantly different (*p* = 0.017). Height was significant in univariate analysis (shown in Supplementary Table 3), but not in multivariate analysis. For distal colon length, only bowel habits were significantly different in univariate analysis (shown in Supplementary Table 3) but not in multivariate analysis.

## Discussion

This study using CT colonography shows that females and constipated (less frequent defecation) patients have a significantly longer colon, and that these trends also are shown in analyses limited to the length of the proximal colon. However, colon length is not associated with physique, regardless of gender. These observations are confirmed by two different statistical methods. To our knowledge, this is the first study to show that bowel habits (defecation frequency) clearly correlate with colon length in a cohort study. A previous case–control study (*n* = 51) utilizing CT colonography reported that patients with functional constipation had a significantly longer colon compared with diarrhea type IBS or control patients [22]. These results suggest that defecation frequency is associated with colon length and are consistent with the results of the present study.

The difference in colon length between “daily” and “constipated” groups was an average of 11 cm and the difference between the “daily” and “intermediate” groups was an average of 7 cm. It is important to consider how such seemingly small difference could affect defecation frequency. A plausible assumption is that constipated bowel habits could lengthen the colon as a result of fecal impaction. According to a recent murine physiology study, however, elongation of colon by longitudinally stretching the colonic muscle has the effect of inhibiting the colonic migrating motor complexes which are responsible for fecal pellet propulsion in the murine large bowel, finally leading to colonic accommodation and slow transit [25]. However, a partially obstructed outlet in a murine colon resulted in an elongated impacted colon with slowed transit [26]. A similar mechanism may be active in the human colon.

In the present study, proximal colon elongation was present while distal colon elongation was not present in patients classified as constipated. In a nuclear scintigraphy study in children, although unable to quantify colon length, transverse colon elongation is more common but sigmoid colon elongation is not present in those with slow-transit constipation [27]. Even in adults, slow transit constipation could influence proximal colon elongation. To diagnose slow transit constipation, objective tests, e.g., daily stool weight (< 35 g/d), colonic transit time, and anorectal function are required, and diagnostic evaluations should be performed while the patient is not taking laxatives [28]. These objective tests may be onerous for patients to undergo. In addition to the Bristol Stool Form Scale which can estimate colonic transit time [29], proximal colon length measured by CT colonography might be a new surrogate biomarker to identify slow transit constipation.

Notably, the present study has clearly demonstrated that the female colon is longer than the male colon, consistent with two previous relatively large-scale cohort studies; one study applied CT colonography to asymptomatic American patients (*n* = 505) [21] and another used barium enema examination for Japanese patients (*n* = 920) [15]. Sex hormones can affect bowel transit time in women during different stages of their menstrual cycle, although they do not appear to have a major effect on bowel function under normal physiological conditions [30]. During childhood, constipation is common in boys, but is much more common in female adults aged 15–50 years, that is, during their reproductive years [30]. In addition, women with severe constipation have a high incidence of having undergone gynecological surgery [30]. In the present study, indeed, female patients had a higher prevalence of undergoing laparotomy and patients with major laparotomy had a trend toward a longer proximal colon. These observations imply that hormonal and/or gynecological changes are associated with bowel habits and may subsequently alter the colon length.

Measurement methods other than CT colonography have individual drawbacks in nature, as mentioned above in the introduction. At present, CT colonography is the most reliable method to measure actual colon length. However, previous studies using CT colonography showed various values for the length of the entire colon, as summarized in Table 5 [17–19, 21, 31]. It is obvious that patients with incomplete colonoscopy have a long colon [17]. The colon in Americans appears longer than in Japanese patients, but we must consider differences in the measurement software used in the USA and Japan. As part of image processing, both software packages (V3D Colon, Viatronix Inc., USA, and AZE Virtual Place, Canon Medical System, Japan) automatically create a centerline that serves as the focal point for three-dimensional fly-through image review. The length of the centerline from the anus to the cecal tip represents the length of the entire colon, but the centerlines automatically generated depend on the software used. For instance, a submerged lumen with fecal fluid and collapsed segments of colon are not identically considered by the different software, and the software is evolving year by year, causing distinct differences. Nagata et al. analyzed the American colon using an open-access database (the Cancer Image Archive) and reported that the total colorectal length in Americans (*n* = 650) was significantly longer than in Japanese (*n* = 650) but the difference was very small [31], which suggests that racial difference in colon length likely do not exist. Considering that colon length was not associated with height in a previous study [21] as well as in the present study, the difference between American and Japanese colon lengths is negligible.

**Table 5:** Reports measuring the entire colon length using CT colonography

First author	Publication year	Software	*N*	Indication	Nationality	Female/male	Age, year (mean ± SD)	Colon length, cm (mean ± SD)	Range
Hanson et al. [17]	2007	Viatronix	100	Incomplete CS	American	59/41	63.4 ± 10.6	210.8 ± 38.2	Maximum > 3 m
			100	Complete CS	American	40/60	58.2 ± 7.9	167.0 ± 20.8	
Duncan et al. [18]	2009	Viatronix	338	Screening	American	122/216	58 ± 7.0	189	75 to 257 cm
Khashab et al. [21]	2009	Viatronix	505	Screening	American	266/239	56.6 ± 7.3	189.5 ± 26.3	120 to 299 cm
Eickhoff et al. [19]	2010	Viatronix	100	Screening	American	40/60	58.2 ± 7.9	167.0 ± 20.8	n/a
Nagata et al. [31]	2013	AZE	650	Screening	American	335/315	58.9	158.2 ± 21.7	n/a
			650		Japanese	321/329	59.6	154.7 ± 20.4	n/a
Present study	2020	AZE	295	FIT positive	Japanese	141/154	58 ± 11.0	150.3 ± 18.5	109.5 to 195.9 cm

Report by Punwani et al. [20] is not cited because data on entire colon length in all patients are not available

*SD* standard deviation, *CS* colonoscopy, *FIT* fecal immunochemical test, *n/a* not available

This study has several acknowledged limitations. First, this is a post hoc analysis using data obtained from a previous multi-center trial, and the sample size was not calculated. Second, all patients were Japanese manifesting positive FIT, and the generalizability of these findings may be limited. Third, we did not apply the Rome IV criteria to assess bowel habits but asked about defecation frequency alone. Accordingly, patients with functional constipation may be classified into a non-constipated group. Fourth, colon length was measured by one author (KU). This could result in a lack of objective analysis because colon length was manually estimated in case of failure of the automatic process.

In conclusion, this study has clearly demonstrated that bowel habits (defecation frequency) and gender both correlate with the length of the entire colon measured by CT colonography, and in particular, the proximal colon. Proximal colon length might be a new surrogate biomarker to objectively diagnose slow transit constipation.

## Supplementary Information

Below is the link to the electronic supplementary material.

Supplementary file1 (DOCX 25 kb)
