# Supplementary material for: Bowel habits and gender correlate with colon length measured by CT colonography
Source: Jpn J Radiol. 2021 Oct 11;40(3):298–307. doi: 10.1007/s11604-021-01204-7 (PMC8891197; doi:10.1007/s11604-021-01204-7)
Supplement: Supplementary file 1 — Supplementary file1 (DOCX 25 kb) [file 11604_2021_1204_MOESM1_ESM.docx]

| Supplementary Table 1. Characteristics of study patients by gender | | | | |
| --- | --- | --- | --- | --- |
|  |  | Male  n=154 | Female  n=141 | *p* value |
| Age, years |  | 58.9 ± 11.0 | 57.1 ± 11.2 | 0.173* |
| Height, cm |  | 167.2 ± 6.5 | 155.0 ± 5.9 | <0.0001* |
| Weight, kg |  | 65.9 ± 10.0 | 54.0 ± 8.7 | <0.0001* |
| Body mass index, kg/m^2^ | | 23.6 ± 3.1 | 22.4 ± 3.4 | 0.0041* |
| History of laparotomy | Major, n (%) | 5 (3.2%) | 10 (7.0%) | 0.013** |
|  | Minor, n (%) | 29 (24%) | 43 (31%) |  |
|  | None, n (%) | 120 (78%) | 88 (62%) |  |
| Bowel habits | Daily, n (%) | 121 (79%) | 68 (48%) | <0.0001** |
|  | Intermediate, n (%) | 25 (16%) | 43 (31%) |  |
|  | Constipated, n (%) | 8 (5.2%) | 30 (21%) |  |
| * one-way ANOVA ** chi square test | |  |  |  |
| Height, weight, and body mass index were significantly greater in males, compared with females. Females had a significantly higher proportion undergoing major surgery and having constipated bowel habits. | | | | |
| Bowel habits were divided into three groups. daily group, everyday; intermediate group, once every two or three days; and constipated group, less than once in three days. | | | | |
|  |  |  |  |  |
| History of Laparotomy: those who underwent gastro- intestinal or uterus resections were classified as major, and others were classified as minor. | | | | |

| \| Supplementary Table 2. Characteristics of patients by bowel habits \| \| \| \|  \|  \| \| --- \| --- \| --- \| --- \| --- \| --- \| \|  \|  \| Daily  n=189 \| Intermediate  n=68 \| Constipated  n=38 \| *p* value \| \| Gender \| Male, n (%) \| 121 (79%) \| 25 (16%) \| 8 (5%) \| <0.0001** \| \|  \| Female, n (%) \| 68 (48%) \| 43 (30%) \| 30 (21%) \|  \| \| Age, year \| Mean ± SD \| 58.2 ± 10.9 \| 56.6 ± 10.9 \| 59.9 ± 11.9 \| 0.32* \| \| Weight, kg \| Mean ± SD \| 61.9 ± 11.7 \| 58.5 ± 9.9 \| 55.0 ± 7.7 \| 0.0006* \| \| Height, cm \| Mean ± SD \| 162.8 ± 8.9 \| 159.8 ± 8.5 \| 157.3 ± 6.0 \| 0.0003* \| \| Body mass index, kg/m^2^ \| Mean ± SD \| 23.2 ± 3.4 \| 22.9 ± 3.3 \| 22.2 ± 2.9 \| 0.20* \| \| History of laparotomy \| Major, n (%) \| 8 (53%) \| 3 (20%) \| 4 (27%) \| 0.50** \| \|  \| Minor, n (%) \| 49 (68%) \| 16 (22%) \| 7 (10%) \|  \| \|  \| None, n (%) \| 132 (63%) \| 49 (24%) \| 27 (13%) \|  \| \| * one-way ANOVA ** chi square test \| \|  \|  \|  \|  \| \| Age, body mass index and a history of laparotomy had no significant differences. In contrast, the proportion of patients with “daily” bowel habits was significantly lower in females compared with males (“daily” vs. “intermediate”: p=p<0.001; “daily” vs. “constipated”: p=p<0.001). The weight of patients with “daily” bowel habits tended to be higher (vs. “intermediate”: p=0.077; vs. “constipated”: p=0.01) and height was significantly greater (vs. “intermediate”: p= 0.036; vs. “constipated”: p= 0.001). \| \| \| \| \| \| \| Bowel habits were divided into three groups. daily group, everyday; intermediate group, once every two or three days; and constipated group, less than once in three days. \| \| \| \| \| \| \|  \| \| History of Laparotomy: those who underwent gastro- intestinal or uterus resections were classified as major, and others were classified as minor. \| \| \| \| \| \|  \|   Supplementary Table 3. Univariate linear regression analysis for variables correlating with colon length | | | | | | | | | |
| --- | --- | --- | --- | --- | --- | --- | --- | --- | --- | --- | --- | --- | --- | --- | --- | --- | --- | --- | --- | --- | --- | --- | --- | --- | --- | --- | --- | --- | --- | --- | --- | --- | --- | --- | --- | --- | --- | --- | --- | --- | --- | --- | --- | --- | --- | --- | --- | --- | --- | --- | --- | --- | --- | --- | --- | --- | --- | --- | --- | --- | --- | --- | --- | --- | --- | --- | --- | --- | --- | --- | --- | --- | --- | --- | --- | --- | --- | --- | --- | --- | --- | --- | --- | --- | --- | --- | --- | --- | --- | --- | --- | --- | --- | --- | --- | --- | --- | --- | --- | --- | --- |
|  | Entire colon | | | Proximal colon | | | Distal colon | | |
|  | coefficients | 95% CI | *p* value | coefficients | 95% CI | *p* value | coefficients | 95% CI | *p* value |
| Age | 0.078 | -0.11 to 0.27 | 0.422 | 0.086 | -0.48 to 0.22 | 0.21 | -0.0075 | -0.13 to 0.11 | 0.89 |
| Gender (M=1, F=2) | 7.2 | 3.0 to 11.3 | 0.001 | 5.4 | 2.5 to 8.3 | < 0.001 | 1.8 | -0.78 to 4.4 | 0.17 |
| Weight | -0.087 | -0.28 to 0.10 | 0.37 | -0.028 | -0.16 to 0.11 | 0.69 | -0.060 | -0.18 to 0.057 | 0.32 |
| Height | -0.23 | -0.47 to 0.017 | 0.069 | -0.18 | -0.35 to -0.009 | 0.039 | -0.047 | -0.19 to 0.10 | 0.54 |
| Body Mass Index | 0.087 | -0.56 to 0.73 | 0.79 | 0.27 | -0.18 to 0.72 | 0.24 | -0.18 | -0.58 to 0.21 | 0.36 |
| History of surgery | 1.2 | -2.4 to 4.9 | 0.51 | 0.46 | -2.1 to 3.1 | 0.73 | 0.77 | -1.5 to 3.0 | 0.50 |
| Bowel habits | 6.0 | 3.1 to 8.9 | < 0.001 | 4.0 | 1.9 to 6.1 | < 0.001 | 1.9 | 0.1 to 3.8 | 0.036 |
| CI: confidence interval, M=Male, F=Female | | | |  |  |  |  |  |  |
| Colon was divided into proximal segment and distal segment at the height of the iliac crest. | | | | | | | | |  |
| History of Laparotomy was classified in major (2), minor (1) and none (0). | | | | | | | | |  |
| Bowel habits was classified in a daily group (1), an intermediate group (2) and a constipated group (3) | | | | | | | | |  |
